# Helminths as architects of trained tolerance: implications for human health

**DOI:** 10.1002/cti2.70086

**Published:** 2026-03-22

**Authors:** Quinn Moroz, Jorge Lucas Nascimento Souza, Neima Briggs

**Affiliations:** ^1^ Department of Immunobiology Yale University School of Medicine New Haven CT USA; ^2^ Laboratory of Immunobiology and Control of Parasites, Department of Parasitology, Institute of Biological Sciences Universidade Federal de Minas Gerais Belo Horizonte MG Brazil; ^3^ Department of Internal Medicine (Infectious Diseases) Yale University School of Medicine New Haven CT USA

**Keywords:** adaptive and innate immunity, allergy, autoimmunity, cancer, coinfection, vaccine responsiveness

## Abstract

Helminths infect nearly 2 billion people worldwide and are a major cause of chronic morbidity in low‐resource regions. Unlike bacterial and viral pathogens that elicit protective memory, helminths actively remodel host immunity to enable their years‐long persistence and reinfection. We put forward the concept of ‘trained tolerance’, which describes the durable yet reversible system‐wide immunological programme helminths exert on the host to promote their own survival. In humans, this altered immune set point shapes host responses to concurrent challenges, including cancer immunity, allergy, autoimmunity, coinfections and vaccine effectiveness. Here, we outline research priorities, including defining species‐specific immune correlates of protection versus tolerance, integrating longitudinal cohorts and controlled human infection studies with single‐cell and spatial profiling, establishing standardised biomarker panels to track immune recalibration after parasite clearance and evaluating the impact of anthelmintic treatment prior to immunisation campaigns to restore germinal centre function and durable protection.

## Introduction

Helminths are parasitic worms that infect every known mammalian species with marked host specificity. Although now concentrated in rural regions of Latin America, Southeast Asia and sub‐Saharan Africa (see Table 1 for epidemiologic summary), human helminth infections were once nearly universal, with archaeological evidence of helminth eggs in Palaeolithic coprolites and descriptions of disease in ancient medical texts.^1^ Today, nearly 2 billion people are infected with at least one helminth, with profound consequences for immune function and human health.^2^

**Table 1:** Major chronic human helminth infections (≥ 1 million prevalence, ≥ 1 year infection)

Species	Common name	Estimated prevalence	High burden regions	Helminth lifespan in host	Route of transmission	Tissue infected	Size (adults)
**Flatworms: Tapeworms**							
*Taenia* spp.	Pork/beef/Asian tapeworm	5.5 million^33^, ^72^	Africa, Asia, Latin America	2–3 years, occasional up to 25 years	Ingestion of raw or undercooked infected meat, faecal‐oral	Primarily intestines, but can involve brain, muscle, skin, eyes	2–7 m
*Hymenolepis nana*, *H. diminuta*	Dwarf/rat tapeworm	75 million^73^	Southern Europe, Russia, India, Latin America, southern United States	4–6 weeks, autoinfection leads to years‐long infections (*H. nana* only)	Faecal‐oral or ingestion of insects containing cysticercoid	Small intestine	15–40 mm (*H. nana*) 200–600 mm (*H. diminuta*)
*Diphyllobothrium latum*	Fish tapeworm^74^	20 million	Northern hemisphere, including Europe, North America and Asia	Up to 25 years	Ingestion of raw or undercooked fish	Intestines	2–15 m
**Flatworms: Flukes**							
*Schistosoma haematobium*, *S. mansoni* and *S. japonicum*	Blood fluke	236 million^75^, ^76^	Sub‐Saharan Africa, South America, Caribbean (*S. mansoni*); Africa and the Middle East (*S. haematobium*); China, Philippines, Sulawesi (*S. japonicum*)	3–10 years	Skin penetration from infected body of water	Liver, spleen, brain (*S. Japonicum, S. mansoni*), spinal cord (*S. mansoni, S. haematobium*), bladder (*S. haematobium*)	6–17 mm
*Fasciola hepatica*, *F. gigantica*	Liver fluke	56 million people with one or more foodborne trematode infection^72^	Andes highlands, Nile Delta of Egypt, parts of Asia and Europe	Up to 13 years	Faecal‐oral	Liver parenchyma and bile ducts; rare ectopic migration to lungs, subcutaneous tissue, pancreas or brain	*F. hepatica*: up to 30 mm, *F. gigantica*: up to 75 mm
*Clonorchis sinesis*, *Opisthorchis* spp.	Liver fluke		Asia, especially South Korea, China, Northern Vietnam	Up to 26 years	Ingestion of raw or undercooked infected freshwater fish	Liver, gallbladder, bile duct	5–25 mm
*Paragonimus westermani*, *Paragonimus* spp.	Lung fluke		Americas, Africa, Southeast Asia, occasionally in Canada and United States	Up to 25 years	Ingestion of infected undercooked or pickled crab or crayfish	Duodenum, peritoneal cavity, lungs, occasional central nervous system or subcutaneous tissue	7–12 mm
**Roundworms: Intestinal**							
*Ascaris lumbricoides* and *A. suum*	Roundworm	732 million^77^	South‐eastern Asia, South America, Eastern Africa, Western Africa and south‐eastern United States	1–2 years	Faecal‐oral	Intestine, lungs	15–49 cm
*Necator americanus, Ancylostoma duodenale* and *A. ceylanicum*	Hookworm	430 million^77^	Africa, Asia, Australia, the Americas, India, Middle East, south‐eastern United States	1–2 years	Skin penetration from infected soil or faecal‐oral (much less common)	Intestine	1–2 mm
*Trichuris trichiura*	Whipworm	800 million^77^	Central Africa, India and Southeast Asia, and south‐eastern United States	1 year	Faecal‐oral	Small intestine, ascending colon	8–13 mm
*Strongyloides stercoralis*	Threadworm	100 million^78^	Mexico, Africa, South America and Southeast Asia, and south‐eastern United States	Up to 25 years through autoinfection	Skin penetration from infected soil	Gastrointestinal tract and lungs (chronic infection); central nervous system, liver, skin (in disseminated cases)	30–50 mm
*Enterobius vermicularis*	Pinworm	200 million^79^	Worldwide	2 months	Faecal‐oral	Intestine, appendix, occasionally the female genital tract	2–13 mm
**Roundworms: Filarial**							
*Onchocerca volvulus*	River blindness	20.9 million^72^	Africa, Latin America, Middle East	Up to 15 years	Black fly bite	Skin, subcutaneous connective tissues	19–50 cm
*Wuchereria bancrofti*, *Brugia* spp.	Lymphatic filariasis	51.4 million^72^	Sub‐Saharan Africa, Western Pacific islands, Southeast Asia	5–7 years	Mosquito bite	Lymphatics, thoracic muscles	13–55 mm
*Loa loa*	African eye worm	20 million^80^	Africa, Yemen	Up to 17 years	Deerfly bite	Peripheral blood, lungs	30–70 mm

This table lists human helminth species with global prevalence greater than 1 million people and typical infections lasting more than 1 year, along with their common names, estimated prevalence, regions with highest burden, infection duration, transmission route, primary tissue tropism and size as adult worms.

Helminths have exerted sustained selective pressure in mammals, shaping the human immune genome with single‐nucleotide polymorphism (SNP) in genes linked to macrophage activation, regulatory T‐cell (Treg) development, leukocyte integrins and inhibitory checkpoints.^3^ Population‐level analyses indicate that parasite pressure has driven selection on several interleukin pathways, particularly those governing type 2 and regulatory responses, such as STAT6 and interleukin 10 (IL‐10), IL‐4 and IL‐13.^4^, ^5^

This coevolution has enabled helminths to persist within hosts for years, often with repeated reinfections. Although partial immunity may limit pathology, sterilising protection is uncommon.^6^ Prior work framed this as ‘deficient acquired immunity’ (DAI), which describes the failure of the adaptive immune system to mount protective memory, which is thought to be the cause of suboptimal vaccine performance seen in helminth‐endemic settings.^7^ Building on this framework, we propose the term ‘trained tolerance’ to describe the complex, system‐wide immunological reconfiguration helminths exert on the host to promote their own survival.

Helminths achieve a state of trained tolerance primarily through their excretory and secretory immunomodulatory molecules, including protease inhibitors, TGF‐β mimics, RNases such as schistosome omega‐1 and immunomodulatory glycans.^8^ Chronic exposure to these factors induces a tolerogenic state across macrophages, mast cells, myeloid suppressor cells, T cells and B cells, with transferable effects demonstrated by adoptive transfer models.^8^, ^9^, ^10^, ^11^

**Figure 1:**
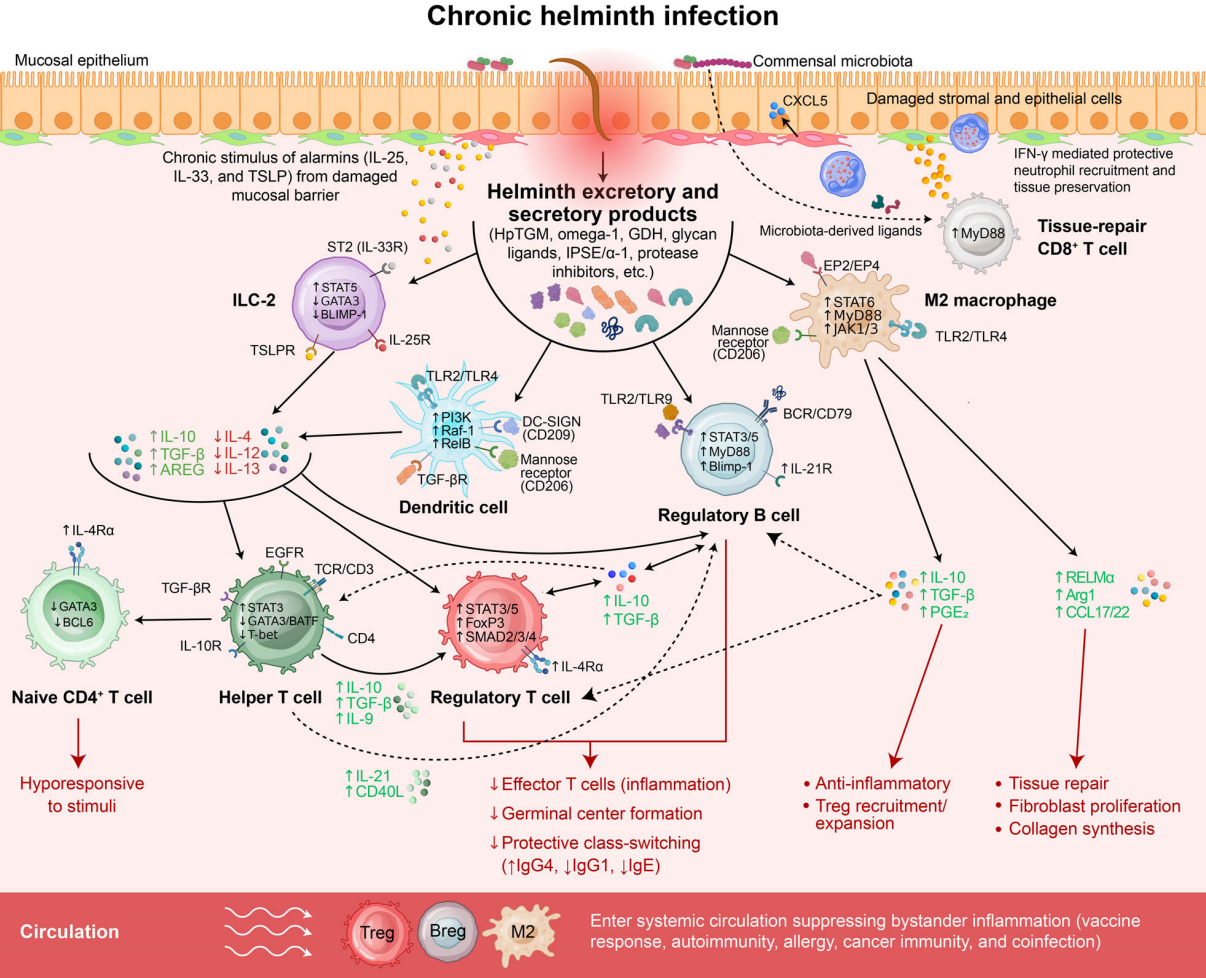
Immune reprogramming by helminths establishes a state of trained tolerance: Chronic helminth infection drives a coordinated reprogramming of barrier, innate and adaptive immunity towards a regulatory state that preserves tissue integrity at the cost of sterilising clearance.^7^ Initial epithelial damage and alarmin release (IL‐25, IL‐33 and TSLP) activate type 2 immunity and stimulate ILC2s but prolonged exposure to alarmins shifts ILC2s towards a regulatory‐leaning phenotype characterised by increased AREG, IL‐10 and TGF‐β to support epithelial repair and Treg expansion.^15^ Concurrent microbiota‐dependent sensing through stromal MyD88 signalling promotes tissue‐protective CD8^+^ T‐cell responses that produce IFN‐γ to recruit neutrophils and prevent barrier breakdown.^16^ Helminth excretory/secretory (E/S) molecules condition antigen‐presenting cells: dendritic cells engaged through TLR2/TLR4, DC‐SIGN and mannose receptors upregulate IL‐10/TGF‐β and reduce IL‐12 via PI3K–Raf‐1–RelB signalling, favoring Treg over Th1/Th17 differentiation.^11^, ^14^ Macrophages polarise to an M2 state through STAT6, PGE2–EP2/EP4 and MyD88‐linked pathways, producing IL‐10, TGF‐β, RELM‐α, Arg1 and CCL17/22 to limit inflammation and promote fibroblast‐mediated repair.^11^, ^15^, ^17^ Regulatory T cells expand and stabilise through IL‐10, TGF‐β, IL‐4Rα and STAT3/5–SMAD2/3/4 signalling, suppressing effector T‐cell responses, while regulatory B cells induced through TLR2/TLR9 and BCR signalling secrete IL‐10/TGF‐β that constrain germinal centre formation and protective class switching.^21^, ^23^, ^26^ Together, these rewired networks raise activation thresholds and establish a systemic ‘trained tolerance’ state, disseminated by circulating Tregs, Bregs, M2 macrophages and regulatory‐skewed ILC2s, with broad consequences for vaccine responses, allergy, autoimmunity, cancer and coinfections.

The established concept of ‘disease tolerance’ broadly refers to host strategies to limit tissue damage at the cost of controlling pathogen burden.^12^ We use the term trained tolerance to denote the imprinting of a durable yet reversible regulatory set point across the innate and adaptive immune system with chronic helminth antigen exposure. Rather than representing an immune deficiency, trained tolerance reflects an actively maintained recalibration of activation thresholds that bias towards IL‐10‐ and TGF‐β–dominant programmes and reshapes host immunity to heterologous challenges (Figure 1). By comparison, ‘trained immunity’ describes durable epigenetic and metabolic reprogramming of innate immune cells that enhances or modifies their responsiveness to subsequent challenge.^13^, ^14^

In this review, we examine the cellular mechanisms that enable trained tolerance and its broad consequences for human health. We conclude with research priorities to define the kinetics of trained tolerance, species‐specific mechanisms and the therapeutic potential of these pathways for limiting pathology in inflammatory diseases.

## Cellular architecture of helminth‐induced trained tolerance

### Early tissue sensing and regulatory priming

Helminths establish chronic infections by gradually diverting host immunity from protective type 2 effector pathways towards regulatory control. Upon tissue invasion, epithelial ‘alarmins’ (IL‐33, IL‐25, TSLP) are rapidly released, initiating a cascade that activates group 2 innate lymphoid cells (ILC2s) and promotes helper T‐cell 2 (Th2) effector differentiation. This early response enhances mucus secretion, smooth muscle contractility and eosinophil recruitment, which act as barrier defence mechanisms that constrain parasite migration and fecundity.^15^ However, sustained exposure to helminth‐derived immunomodulatory excretory and secretory products actively instructs expansion of regulatory circuits initiated at primary entry sites of helminth invasion, typically the skin or mucosa. This coordinated reprogramming dampens innate effector function and constrains the development of adaptive immunity.

### Stromal and innate immune rewiring

Emerging data is challenging the strict Th1 versus Th2 paradigm of protection to helminth infections. Notably, Westfall *et al*.^16^ recently demonstrated that the interferon (IFN)‐γ acts on intestinal stromal cells to promote neutrophil recruitment during helminth infection, and that this response is initiated by MyD88‐dependent sensing of the microbiota, linking microbial cues to a tissue‐protective, tolerance‐oriented immune circuit. These findings show that the protective and tolerant arms of immunity can intersect and sometimes cooperate in order to maintain host integrity during infection. Other mechanisms once thought of as purely defensive, such as Th2‐driven tissue repair, IL‐10 secretion or antibody class switching, may serve dual roles, simultaneously limiting tissue damage while enabling parasite persistence.

Innate immune cells also change during infection and promote a systemic anti‐inflammatory state. In *Heligmosomoides polygyrus*, a helminth‐secreted glutamate dehydrogenase epigenetically rewires macrophage pathways to drive prostaglandin E2 (PGE2)‐centred immunoregulation that blunts host defence and promotes chronic infection in mice.^17^ In human lymphatic filariasis, monocytes fail to upregulate toll‐like receptors (TLRs) or cytokine production after stimulation.^18^ A recent review by Doolan *et al*.^11^ summarises how helminth antigens can condition dendritic cells and macrophages into hyporesponsive states that compromise T‐cell help and antibody maturation, and propose strategies to re‐prime innate cell memory as an alternative anthelminthic vaccine strategy.

### T‐cell reprogramming

Chronic helminth antigens also dampen T‐cell function beyond classical regulation. In rodent filariasis (*Litomosoides sigmodontis*), parasite‐specific Th2 cells exhibit reduced proliferation and cytokine production via the PD‐1/PD‐L2 coinhibitory pathway. This defect arises independently of Foxp3^+^ Tregs and IL‐10, and unlike classical exhaustion in chronic viral infection, blocking PD‐1 *in vivo* partially restores Th2 cell function and improves worm clearance.^19^ Beyond antigen‐experienced cells, helminth infection reshapes the transcriptional heterogeneity of the naive CD4^+^ T‐cell pool. In mice infected with rodent hookworm (*Nippostrongylus brasiliensis*), single‐cell RNA sequencing demonstrated a contraction of quiescent and type I interferon–associated naïve clusters with the expansion of a unique helminth‐associated naïve cluster marked by elevated *Il4ra* and canonical IL‐4–induced genes, including *Pros1*, *Lgals3* and *Socs1*. These changes were IL‐4 dependent and associated with reduced proliferation and effector differentiation following heterologous antigen challenge by vaccination. Following helminth clearance, the IL‐4–responsive cluster contracted and naïve cluster proportions trended back towards baseline.^9^

Regulatory T cells are the most thoroughly defined protolerogenic population in helminth‐driven tolerance. Helminths can expand Foxp3^+^ Tregs through excreted and secreted products, such as the *H. polygyrus* TGF‐β mimic (Hp‐TGM), and indirectly by conditioning dendritic cells and macrophages to adopt tolerogenic phenotypes that favor regulatory T‐cell differentiation and support parasite persistence while limiting tissue pathology.^20^ Across natural and experimental infections, these helminth‐elicited Tregs also suppress bystander inflammation in allergy and autoimmunity, pointing to molecular pathways that could be developed as anti‐inflammatory therapies.^21^ In a recent 2‐year controlled human hookworm infection study, first‐in‐life infections of adult volunteers induced early expansion of CD45RA^+^ Tregs correlating with the establishment of chronic infection. However, compared with individuals living in endemic settings, these Tregs exhibited lower expression of functional markers, including inducible T‐cell costimulator (ICOS), tumor necrosis factor receptor 2 (TNFR2) and latency‐associated peptide (LAP) and reduced suppressive capacity *in vitro*, suggesting that full functional maturation of helminth‐induced regulation may come with early in life or repeated exposures.^22^

### Bregs and humoral regulation

In addition to Tregs, regulatory B cells (Bregs) have emerged as an important contributor to helminth‐driven immune tolerance. Across multiple helminth models, infection expands B cell subsets with regulatory function, most prominently via IL‐10 secretion, capable of suppressing effector T‐cell responses and limiting tissue inflammation, thereby favoring parasite persistence rather than clearance.^23^, ^24^, ^25^ In schistosomiasis, peripheral B cells exhibit enhanced IL‐10 production, particularly within the CD1d^hi^ subset, and increased expression of regulatory markers including LAP/TGF‐β, correlating with diminished effector T‐cell responses.^26^ In a cohort of patients with multiple sclerosis, purified CD19^+^ B cells from helminth‐infected individuals produced increased IL‐10 and exhibited higher CD1d expression, suppressing myelin‐specific T‐cell proliferation and IFN‐γ production *in vitro*. These regulatory effects were not observed in multiple sclerosis patients with other chronic infections, including *Trypanosoma cruzi* or *Paracoccidioides brasiliensis*, supporting a helminth‐specific induction of regulatory B cell function associated with disease activity.^27^ Although conclusive evidence of durable epigenetic reprogramming in helminth‐induced Bregs is still emerging, the accumulating literature supports a Breg axis of regulation that complements Treg‐ and myeloid‐centred tolerogenic networks.^24^

In endemic settings, patterns of antibody responses to helminths are more often associated with infection intensity than predictive of effective parasite elimination. In human schistosomiasis (*S. japonicum* and *S*. *mansoni*), a higher parasite‐specific IgE:IgG4 ratio has been associated with lower risk of reinfection, whereas elevated IgG4 (and relatively lower IgE) correlates with heavier burdens and increased susceptibility. The noninflammatory nature of IgG4, capable of blocking IgE‐mediated effector pathways, may reduce bystander tissue damage. Recent reviews emphasise that in chronic helminth exposure, tissue‐resident type 2 responses and regulatory networks, including humoral subsets, may contribute more to coexistence than to protective immunity.^20^

### Persistence and reversibility of trained tolerance

A central unresolved question in helminth immunology is whether the regulatory state established during chronic infection represents a long‐lived immune imprint or a condition that requires ongoing parasite exposure to be maintained. Although animal models demonstrate that helminth infection can generate long‐lived plasma cells and memory Th2 responses capable of rapid recall, epidemiologic data in humans suggest that repeated exposure does not reliably reduce prevalence or parasite burden with age.^6^, ^28^ This apparent disconnect suggests that immune exposure in endemic settings is redirected towards a regulatory equilibrium rather than sterilising protection.

Insights into the persistence of trained tolerance in humans derive largely from treatment studies that assess immune recalibration after parasite clearance. In hookworm and *S. haematobium* infections, Treg frequency and suppressive activity normalise around 3 months post anthelminthic therapy.^29^, ^30^ Helper T‐cell polarisation also trends back towards baseline within months after treatment of schistosomiasis or strongyloidiasis.^31^, ^32^ This limited evidence suggests that trained tolerance requires persistent antigenic stimulation and is therefore reversible, but the kinetics of immune recalibration may depend on the helminth species, exposure intensity and coexisting host factors.^29^, ^30^, ^31^, ^32^ The scarcity of longitudinal, high‐resolution human studies limits conclusions about the true durability of trained tolerance.

## Consequences of trained tolerance for human health

**Figure 2:**
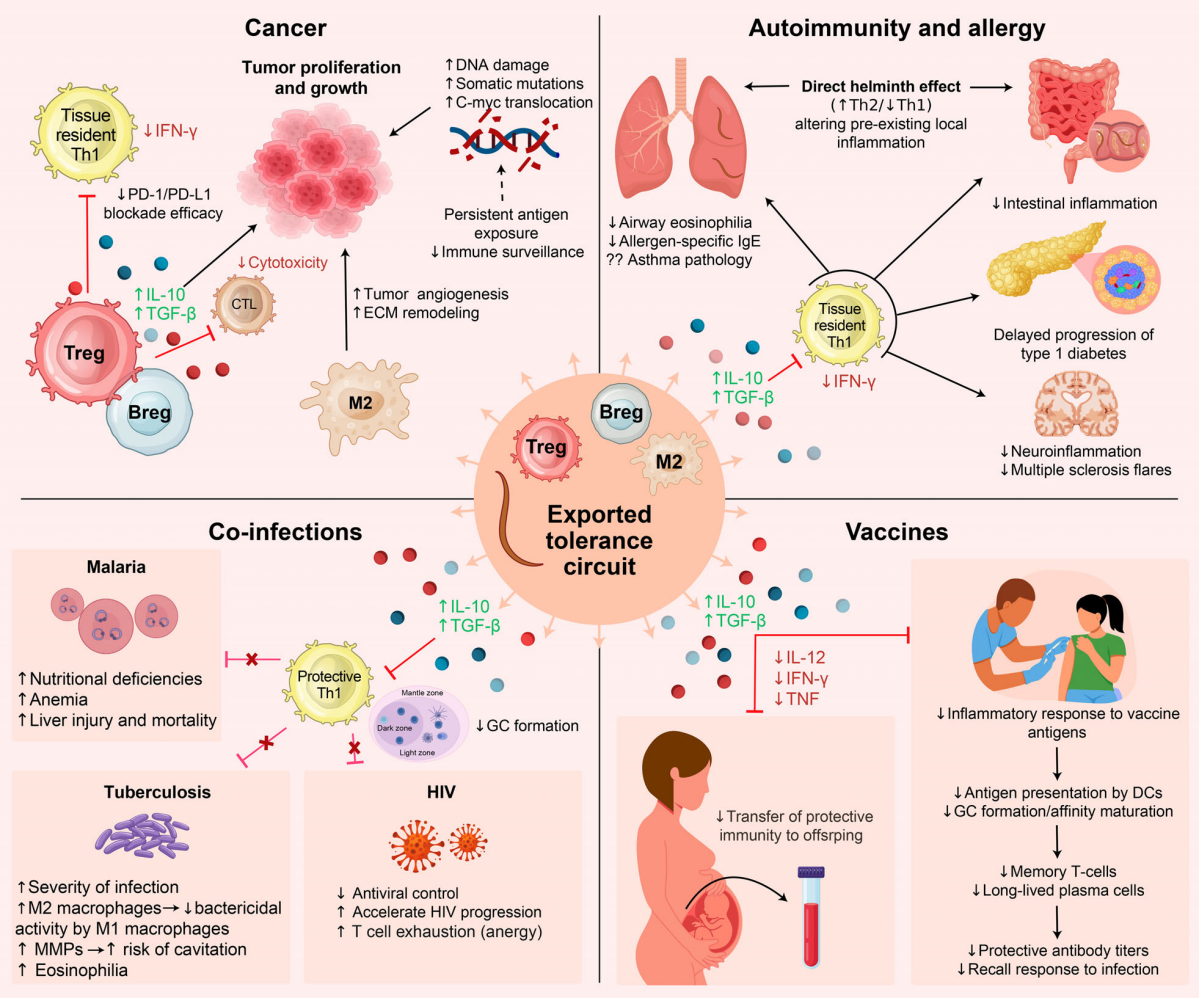
Systemic consequences of trained tolerance on human health: Chronic helminth infection induces tolerogenic circuits that extend beyond mucosal sites, as Tregs, Bregs and M2 macrophages enter the circulation and recalibrate systemic immunity.^21^, ^26^ This ‘exported tolerance’ elevates activation thresholds, suppresses effector priming and alters responses to unrelated antigens, vaccines, tumors and pathogens. In cancer, increased IL‐10 and TGF‐β, regulatory myeloid skewing, and impaired Th1 and cytotoxic T‐cell activity weaken tumor immune surveillance, reduce responsiveness to PD‐1/PD‐L1 blockade, and promote angiogenesis and extracellular matrix remodelling through M2‐associated mediators, such as Arg1, RELM‐α and CCL17/22.^33^, ^42^ Certain helminths can additionally induce DNA damage and genomic instability, contributing to carcinogenesis.^33^, ^40^ In autoimmunity and allergy, dominant regulatory networks suppress allergen‐specific IgE and eosinophilia, attenuate Th1/Th17‐driven autoimmunity, and reduce neuroinflammation and disease flares in multiple sclerosis and type 1 diabetes, although their contributory role in asthma and their therapeutic value from helminth trials show inconsistent clinical benefits.^23^, ^24^, ^27^, ^48^, ^49^, ^51^ During coinfection, tolerance impairs pathogen‐specific Th1 immunity, disrupts germinal centre responses, limits macrophage microbicidal function and accelerates T‐cell exhaustion, exacerbating outcomes in tuberculosis, HIV, and malaria and worsening anaemia and tissue pathology.^54^, ^55^, ^58^, ^59^, ^60^, ^63^, ^65^ Vaccination responses are similarly blunted, as reduced IL‐12, IFN‐γ and TNF during priming impair dendritic cell activation, Tfh differentiation and germinal centre formation, limiting high‐affinity class‐switching and long‐lived plasma cell development, with recovery of immune response after deworming.^66^, ^71^ Maternal helminth infection further diminishes passive antibody transfer of vaccine‐mediated immunity and leads to reduced infant protection against infectious challenges.^69^, ^70^

The concept of trained tolerance provides a framework to understand how helminth infection may influence diverse immunological challenges, including cancer immunity, autoimmunity, allergic disease, vaccine responsiveness and outcomes of coinfection (Figure 2), each of which is examined in detail below.

### Cancer immunity and tumor surveillance

Cancer is often framed as a disease of smoking, pollutants, diet and obesity, yet infectious agents are increasingly recognised as important drivers of carcinogenesis. Within this context, chronic helminthiasis is emerging as both a contributor to cancer risk and a modifier of anti‐tumor immunity. The International Agency for Research on Cancer (IARC) currently designates three helminths as definite human carcinogens (Class 1), namely the liver flukes *Clonorchis sinensis* and *Opisthorchis viverrini* that cause cholangiocarcinoma, and the blood fluke *Schistosoma haematobium* that causes bladder cancer.^33^ Beyond these established associations, human observational studies have linked *S. mansoni* to hepatic carcinomas^34^ and *S. japonicum* to hepatocellular and colorectal cancers.^35^ Animal studies further describe the tumor‐promoting effects of helminths, such as *Toxocara canis* in breast cancer models^36^, ^37^ and *Trichuris spp*. in intestinal neoplasia^38^ (emerging helminth–cancer associations are summarised in Table 2).

**Table 2:** Helminth‐associated cancers beyond IARC Class 1 species

Helminth	Cancer	Proposed oncogenic mechanism
*Echinococcus granulosus*	Breast cancer	*E. granulosus* infection may influence cancer progression by modulating systemic T‐cell responses. Experimental murine coinfection with hydatid cysts and breast tumors increased liver metastases, associated with elevated CD4^+^ T cells but reduced Th1 (IFN‐γ^+^ and CCR5^+^) responses and increased CD25^+^ regulatory T cells, suggesting that echinococcosis creates an immunosuppressive environment favoring tumor metastasis^81^
*Schistosoma mansoni*	Hepatocellular carcinoma	Murine infection with *S. mansoni* enhances the carcinogenic effects of diethylnitrosamine, accelerating the onset and increasing the grade of hepatic dysplasia (SCD). While mice exposed to diethylnitrosamine alone (Group I) developed late and mild dysplastic changes, mice in Group II (diethylnitrosamine and infection) exhibited earlier and more severe SCD. This effect was accompanied by earlier elevations in serum alpha‐fetoprotein and ferritin levels in Group II compared to Group I, indicating accelerated activation of tumor‐related mechanisms. Infection alone (Group III) induced granulomas and mild portal fibrosis without dysplasia, while the control group remained normal. These findings suggest that *S. mansoni* amplifies the progression of pre‐neoplastic lesions, creating a hepatic environment more susceptible to carcinogenesis^82^
*Schistosoma japonicum*	Colorectal cancer	In mouse and human studies, *S. japonicum* infection has been found to promote colorectal cancer through persistent inflammation triggered by egg deposition, causing epithelial hyperplasia, DNA damage and increased susceptibility to mutations. Parasite antigens and iNOS contribute to genotoxic stress, while expansion of immunosuppressive cells, including CD4^+^CD25^+^ Tregs and myeloid‐derived suppressor cells (MDSCs), suppresses anti‐tumor immunity and favors tumor progression. Coinfection with bacteria may further enhance tumorigenesis, and molecular alterations such as p53 mutations have been observed in schistosomiasis‐associated colorectal cancer^83^
*Trichuris spp*.	Colon cancer	*T. muris* infection promotes intestinal neoplasia through multiple mechanisms. In wild‐type mice, persistent Th1‐driven inflammation (increased IFN‐γ, TNF‐α and IL‐6) led to epithelial hyperplasia, aberrant crypt formation and higher neoplasia scores, comparable to those induced by azoxymethane. In APC^min/+^ mice, infection enhanced adenoma development, particularly in the distal small intestine, with a marked increase in the number of small tumors, despite no direct effect on epithelial proliferation in this site. Importantly, chronic infection expanded CD4^+^CD25^+^Foxp3^+^ Tregs and blocking Tregs with anti‐CD25 antibodies abrogated the infection‐driven tumor promotion, highlighting the key role of regulatory pathways in immune evasion and tumor progression^38^
*Toxocara canis*	Breast cancer	In mice, experimental *T. canis* infection promoted 4 T1 tumor growth by shifting the immune response towards a Th2/regulatory profile (highest levels of IL‐4 and IL‐10), reducing cytotoxic CD8^+^ T‐cell activity, increasing proliferation (↑Ki‐67), suppressing apoptosis and DNA repair (↓P53 expression), promoting a pro‐inflammatory microenvironment and stimulating angiogenesis (↑VEGF)^36^, ^45^
*Heligmosomoides polygyrus* (hookworm model)	Colorectal cancer	Murine *H. polygyrus* infection exerts stage‐dependent effects on colitis‐associated colorectal cancer (CAC). When administered during tumor progression, infection did not significantly change tumor growth or distribution. However, infection at the early inflammatory phase markedly exacerbated CAC development by amplifying colonic inflammation. This was associated with long‐lasting immune alterations, including reduced frequencies of CD8^+^ effector T cells and enhanced release of IL‐6 and CXCL1, fostering a pro‐tumorigenic microenvironment. These findings suggest that helminth‐based therapies for IBD, although promising, may have tumor‐promoting side effects under certain conditions^84^

This table summarises helminth species associated with cancer development outside the IARC Class 1 carcinogens, the tumor types reported and the proposed oncogenic mechanisms.

Mechanistically, helminths do not appear to function as classical oncogenic pathogens that directly transform host cells. Rather, both human studies and animal models indicate helminths impair tumor surveillance through sustained immune modulation. Parasite‐driven regulatory cytokine networks and altered myeloid and lymphoid function can impair anti‐tumor immunity. In murine tumor models, helminth‐induced type 2 skewing and expansion of regulatory myeloid cells suppress cytotoxic type 1 pathways required for tumor control.^39^, ^40^ In humans, elevated TGF‐β and IL‐10 signalling, hallmarks of helminth trained tolerance, promote the exclusion of effector T cells from the tumor microenvironment and limit therapeutic responsiveness to PD‐1/PD‐L1 checkpoint blockade.^41^

In addition to immune modulation, chronic helminth infection may promote tumorigenesis through inflammation‐associated genomic stress. Persistent tissue injury, oxidative stress and a dysregulated repair response create environments permissive to mutagenesis and malignant progression.^42^ For example, *Taenia solium* antigens induce DNA damage in the lymphocytes of neurocysticercosis patients by provoking chromosomal translocation event.^43^, ^44^ In a murine tumor model, *T. canis* infection promotes tumor progression, accompanied by reduced CD8^+^ activity, increased IL‐4 and IL‐10 production, and downregulation of p53 tumor suppressor protein.^36^, ^45^

Together, these findings indicate that helminths can contribute to oncogenesis through immune modulation, tissue remodelling and inflammation‐associated genomic instability. In humans, causal links are firmly established for three species classified as Group 1 carcinogens, whereas associations for additional helminths remain epidemiologic or mechanistically inferred. Experimental models nonetheless demonstrate that helminth‐driven regulatory cytokine networks and myeloid skewing can compromise tumor surveillance and foster microenvironments permissive to tumor progression. These effects may be particularly consequential in low‐resource settings where helminth burden intersects with limited access to cancer screening, diagnosis and treatment. Rigorous longitudinal human studies are therefore needed to define the extent to which helminth‐induced immune remodelling contributes to cancer risk and progression.

### Allergy and autoimmune disease

One influential branch of the ‘hygiene hypothesis’ proposes that reduced exposure to helminths alters early‐life immune education, predisposing to allergy and autoimmunity. Although helminths have shaped the human immune system, they are not benign ‘missing friends’. Rather, they are potent immunomodulators capable of reshaping immune responses in ways that may secondarily benefit coexisting inflammatory conditions.^1^ However, attempts to harness this therapeutically have proven difficult. Clinical trials using live helminth exposure for autoimmune conditions have produced limited or inconsistent benefit, underscoring how different species, dosing, timing, helminth pre‐exposure and host factors may confound the immunological response.^46^

In experimental models, helminths reduce allergen‐specific IgE and airway eosinophilia and increase IL‐10 with expansion of regulatory T and B cells.^26^, ^47^ Similarly, injection of excretory/secretory products or recombinant proteins from *S. japonicum* or *T. spiralis* can reduce airway inflammation by limiting eosinophil infiltration and upregulating regulatory cytokines.^24^ These effects reflect not just momentary suppression but reprogramming of myeloid and lymphoid compartments consistent with trained tolerance.

Experimental and clinical observations in autoimmune disease provide some of the strongest evidence that helminths can modulate pathogenic inflammation. Controlled infections with *Trichuris suis* or *Necator americanus* in patients with Crohn's disease increase Tregs and IL‐10 and suppress pathogenic Th1 inflammation.^48^, ^49^ Similar protective effects have been observed in colitis models, where helminth infections dampen intestinal inflammation through IL‐4 and IL‐10 induction and lowered IFN‐γ.^50^ In type 1 diabetes, *S. mansoni*, *H. polygyrus* or *T. spiralis* can delay disease progression through regulatory enhancement.^51^ Together, these findings provide strong evidence that helminth‐induced regulatory pathways can limit pathological inflammation. Efforts are underway to translate them into safe, defined therapeutics rather than live parasitic infections.^52^

### Coinfections

Given the chronicity of helminth infections, coinfection with other pathogens is frequent.^53^, ^54^ These coinfections recalibrate host immunity and often worsen clinical outcomes. By skewing immune responses towards regulatory pathways, helminths dampen protective type 1 immunity, facilitating persistence of pathogens, such as tuberculosis, HIV and malaria.^55^ While these ‘big three’ infections are emphasised because of global impact, similar dynamics are emerging in other diseases, such as recently described with helminth–leprosy coinfection.^56^

In HIV, helminth coinfection skews immunity towards Th2 and regulatory cytokines, drives T‐cell anergy and sustains chronic immune activation, which together weakens antiviral control and can accelerate disease progression.^57^ Epidemiological studies confirm high rates of helminth‐HIV coinfection, particularly in sub‐Saharan Africa.^54^ Advanced immunosuppression with reduced CD4^+^ T‐cell counts or function, as seen in both HIV or HTLV‐1 infections, is associated with more severe and persistent strongyloidiasis, including hyperinfection syndromes.^57^ Importantly, some trials suggest that deworming may improve HIV outcomes by lowering viral load and slowing disease progression, although the overall evidence remains limited.^58^

In tuberculosis, helminth coinfection is strongly associated with an increased risk of active pulmonary disease, with risk rising in proportion to helminth burden.^59^ In Venezuelan children, *Ascaris* and *Trichuris* infections reduced systemic type 1 cytokines and tuberculin skin test reactivity, suggesting suppression of MTB‐specific immunity.^60^ Anthelmintic treatment partially reversed this effect, reducing eosinophilia and circulating IL‐10. In a murine tuberculosis challenge model, chronic *S. mansoni* infection reduces BCG‐mediated protection.^61^ Such findings may have major implications for BCG vaccine efficacy and tuberculosis control in co‐endemic regions.^53^

Similar immune alteration is observed in malaria, where coinfection can exacerbate disease severity. Hookworm‐related anaemia and malnutrition may synergise with Plasmodium infection to worsen outcomes and possibly enhance malaria transmission.^62^ In experimental models, *Plasmodium berghei*–*Ascaris suum* coinfection accelerates pulmonary pathology and mortality, while hookworm animal models of *H. polygyrus* or *N. brasiliensis* coinfection exacerbate malaria‐associated liver injury and mortality.^63^, ^64^, ^65^

Overall, these observations highlight helminth coinfection as a major yet underappreciated determinant of infectious disease outcome. By rewiring immune homeostasis, helminths influence not only pathogen persistence but also the performance of vaccines and therapeutics. Unravelling these complex interactions at molecular and population levels remains an important frontier for improving global health in helminth‐endemic regions.

### Vaccine responsiveness

Helminth infections blunt both the magnitude and durability of vaccine responses. A recent systematic review and meta‐analysis of clinical and animal studies by Natukunda *et al*.^66^ found that helminth exposure significantly diminishes vaccine responses but was reversible with helminth eradication, although the magnitude of this effect varied across pathogens and settings. Helminth‐driven immune reprogramming may elevate the threshold for T‐cell activation, impair germinal centre formation and reduce the production of protective antibody isotypes.

In humans, the most conclusive evidence of helminths reducing response is seen with live vaccines. Helminth‐infected individuals mount weaker cellular and humoral immunity to BCG and measles vaccines than uninfected peers.^66^ Hepatitis B and tetanus, both non‐live vaccines, have also been found to reduce the duration of protective antibodies when infected with *S. mansoni*.^66^ In mice chronically infected with *Taenia crassiceps*, antibody responses to both pneumococcal conjugate and polysaccharide vaccines are impaired.^67^ Complementing these observations, an *S. japonicum* mouse model demonstrated that reduced hepatitis B antibody responses recover partially after anthelmintic treatment.^68^

Maternal infection may further complicate helminth–host interactions by reducing the transfer of protective immunity to offspring. In murine schistosomiasis, offspring exhibited impaired germinal centre responses to tetanus immunisation and a reduction in memory B cell and long‐lived plasma cell generation.^69^ In humans, children born to vaccinated *S. mansoni*–infected mothers had lower anti‐measles IgG levels by age two than children of uninfected mothers, consistent with prenatal helminth exposure influencing vaccine responsiveness.^70^

Whether deworming reliably restores vaccine performance remains unsettled. As highlighted by Natukunda *et al*., benefits appear inconsistent across studies, with a subsequent narrative review reaching a similar conclusion that improvements appear to depend on the parasite species, vaccine platform, and the interval between parasite clearance and immunisation.^66^, ^71^ Together, these findings underscore the challenges introduced by helminth infections in controlling other infectious diseases, particularly by compromising vaccine efficacy. Key gaps include the time course of immune recalibration after treatment, which vaccines are most affected, and how to time mass drug administration efforts relative to immunisation campaigns.

## Concluding remarks

Helminths persist not because the host fails to respond but because the parasites reshape immunity in ways that favor their long‐term survival. The concept of trained tolerance offers a useful framework to understand how a largely protective type 2 response can be redirected into a regulatory state in both the innate and adaptive compartments, suppressing inflammation and enabling parasite persistence. This shift compromises vaccine performance, modulates allergy and autoimmune diseases, reshapes cancer immunity and complicates host control of coinfections.

Despite clear evidence that robust type 2 responses can limit infection burden, sterilising control in response to helminth infection is uncommon. Future human studies should aim to resolve species‐specific patterns of protection and tolerance and the features they share. Longitudinal cohorts and controlled human infection studies should be paired with standardised biomarker panels, single‐cell and spatial profiling, and functional assays that capture rare circulating populations. A key objective is to track the timing of recalibration after parasite clearance, what persists, and which elements are amenable to targeted intervention. Trials should evaluate coordinated use of anthelmintics with vaccination and approaches that restore effective germinal centre and effector responses.

Achieving these goals requires aligning scientific progress with global health priorities. Populations living in helminth‐endemic regions deserve more than experimental insights, as they require access to modern sanitation, reliable diagnostics, context‐specific deworming programmes and immunisation schedules designed with trained tolerance in mind. Reframing helminths as architects of trained tolerance not only clarifies their far‐reaching clinical consequences but also highlights an opportunity to study the complex underlying mechanisms of immune regulation that could inform new strategies for managing inflammatory diseases.

## Author contributions

**Quinn Moroz:** Data curation; visualisation; writing—original draft; writing—review and editing. **Jorge Lucas Nascimento Souza:** Data curation; visualisation; writing—original draft; writing—review and editing. **Neima Briggs:** Conceptualisation; methodology; data curation; supervision; funding acquisition; visualisation; resources; writing—original draft; writing—review and editing.

## Conflict of interest

The authors declare no conflict of interest.

## Ethic statement

Not applicable.

## Data Availability

Data sharing is not applicable to this article as no datasets were generated or analysed during the current study.

## References

[1] Briggs N , Weatherhead J , Sastry KJ , Hotez PJ . The hygiene hypothesis and its inconvenient truths about helminth infections. PLoS Negl Trop Dis 2016; 10: e0004944. 27632204 10.1371/journal.pntd.0004944 PMC5025185

[2] Wright JE , Werkman M , Dunn JC , Anderson RM . Current epidemiological evidence for predisposition to high or low intensity human helminth infection: a systematic review. Parasit Vectors 2018; 11: 65. 29382360 10.1186/s13071-018-2656-4 PMC5791198

[3] Fumagalli M , Pozzoli U , Cagliani R *et al*. The landscape of human genes involved in the immune response to parasitic worms. BMC Evol Biol 2010; 10: 264. 20807397 10.1186/1471-2148-10-264 PMC2940816

[4] Fumagalli M , Pozzoli U , Cagliani R *et al*. Parasites represent a major selective force for interleukin genes and shape the genetic predisposition to autoimmune conditions. J Exp Med 2009; 206: 1395–1408. 19468064 10.1084/jem.20082779 PMC2715056

[5] Mewamba EM , Noyes H , Tiofack AAZ *et al*. Association between polymorphisms of IL4, IL13, IL10, STAT6 and IFNG genes, cytokines and immunoglobulin E levels with high burden of *Schistosoma mansoni* in children from schistosomiasis endemic areas of Cameroon. Infect Genet Evol 2023; 111: 105416. 36889485 10.1016/j.meegid.2023.105416 PMC10167540

[6] Briggs N , Versteeg L , Mejia R *et al*. A Honduran prevalence study on soil‐transmitted helminths highlights serological antibodies to Tm‐WAP49 as a diagnostic marker for exposure to human *Trichuriasis* . Am J Trop Med Hyg 2025; 112: 1017–1025. 39933187 10.4269/ajtmh.24-0514 PMC12062693

[7] McSorley HJ , Maizels RM . Helminth infections and host immune regulation. Clin Microbiol Rev 2012; 25: 585–608. 23034321 10.1128/CMR.05040-11 PMC3485755

[8] Maizels RM , Smits HH , McSorley HJ . Modulation of host immunity by helminths: the expanding repertoire of parasite effector molecules. Immunity 2018; 49: 801–818. 30462997 10.1016/j.immuni.2018.10.016 PMC6269126

[9] Even Z , Meli AP , Tyagi A *et al*. The amalgam of naive CD4(+) T cell transcriptional states is reconfigured by helminth infection to dampen the amplitude of the immune response. Immunity 2024; 57: 1893–1907. 39096910 10.1016/j.immuni.2024.07.006 PMC11421571

[10] Wilson MS , Taylor MD , O'Gorman MT *et al*. Helminth‐induced CD19+CD23hi B cells modulate experimental allergic and autoimmune inflammation. Eur J Immunol 2010; 40: 1682–1696. 20306466 10.1002/eji.200939721 PMC3179601

[11] Doolan R , Putananickal N , Tritten L , Bouchery T . How to train your myeloid cells: a way forward for helminth vaccines? Front Immunol 2023; 14: 1163364. 37325618 10.3389/fimmu.2023.1163364 PMC10266106

[12] Medzhitov R , Schneider DS , Soares MP . Disease tolerance as a defense strategy. Science 2012; 335: 936–941. 22363001 10.1126/science.1214935 PMC3564547

[13] Netea MG , Domínguez‐Andrés J , Barreiro LB *et al*. Defining trained immunity and its role in health and disease. Nat Rev Immunol 2020; 20: 375–388. 32132681 10.1038/s41577-020-0285-6 PMC7186935

[14] Netea MG , Joosten LA , Latz E *et al*. Trained immunity: a program of innate immune memory in health and disease. Science 2016; 352: aaf1098. 27102489 10.1126/science.aaf1098 PMC5087274

[15] Gause WC , Wynn TA , Allen JE . Type 2 immunity and wound healing: evolutionary refinement of adaptive immunity by helminths. Nat Rev Immunol 2013; 13: 607–614. 23827958 10.1038/nri3476 PMC3789590

[16] Westfall S , Gentile ME , Olsen TM *et al*. A type 1 immune‐stromal cell network mediates disease tolerance against intestinal infection. Cell 2025; 188: 3135–3151.e3122. 40267906 10.1016/j.cell.2025.03.043

[17] Bohnacker S , Henkel FDR , Hartung F *et al*. A helminth enzyme subverts macrophage‐mediated immunity by epigenetic targeting of prostaglandin synthesis. Sci Immunol 2024; 9: eadl1467. 39642243 10.1126/sciimmunol.adl1467

[18] Babu S , Blauvelt CP , Kumaraswami V , Nutman TB . Diminished expression and function of TLR in lymphatic filariasis: a novel mechanism of immune dysregulation. J Immunol 2005; 175: 1170–1176. 16002719 10.4049/jimmunol.175.2.1170

[19] van der Werf N , Redpath SA , Azuma M , Yagita H , Taylor MD . Th2 cell‐intrinsic hypo‐responsiveness determines susceptibility to helminth infection. PLoS Pathog 2013; 9: e1003215. 23516361 10.1371/journal.ppat.1003215 PMC3597521

[20] Vacca F , Le Gros G . Tissue‐specific immunity in helminth infections. Mucosal Immunol 2022; 15: 1212–1223. 35680972 10.1038/s41385-022-00531-w PMC9178325

[21] White MPJ , McManus CM , Maizels RM . Regulatory T‐cells in helminth infection: induction, function and therapeutic potential. Immunology 2020; 160: 248–260. 32153025 10.1111/imm.13190 PMC7341546

[22] Manurung MD , Sonnet F , Hoogerwerf MA *et al*. Controlled human hookworm infection remodels plasmacytoid dendritic cells and regulatory T cells towards profiles seen in natural infections in endemic areas. Nat Commun 2024; 15: 5960. 39013877 10.1038/s41467-024-50313-0 PMC11252261

[23] Gao X , Ren X , Wang Q *et al*. Critical roles of regulatory B and T cells in helminth parasite‐induced protection against allergic airway inflammation. Clin Exp Immunol 2019; 198: 390–402. 31397879 10.1111/cei.13362 PMC6857085

[24] Hussaarts L , van der Vlugt LE , Yazdanbakhsh M , Smits HH . Regulatory B‐cell induction by helminths: Implications for allergic disease. J Allergy Clin Immunol 2011; 128: 733–739. 21684587 10.1016/j.jaci.2011.05.012

[25] Reyes JL , Wang A , Fernando MR *et al*. Splenic B cells from *Hymenolepis diminuta*‐infected mice ameliorate colitis independent of T cells and via cooperation with macrophages. J Immunol 2015; 194: 364–378. 25452561 10.4049/jimmunol.1400738

[26] van der Vlugt LE , Zinsou JF , Ozir‐Fazalalikhan A *et al*. Interleukin 10 (IL‐10)‐producing CD1dhi regulatory B cells from *Schistosoma haematobium*‐infected individuals induce IL‐10‐positive T cells and suppress effector T‐cell cytokines. J Infect Dis 2014; 210: 1207–1216. 24795476 10.1093/infdis/jiu257

[27] Correale J , Farez M , Razzitte G . Helminth infections associated with multiple sclerosis induce regulatory B cells. Ann Neurol 2008; 64: 187–199. 18655096 10.1002/ana.21438

[28] Lo NC , Bogoch II , Blackburn BG *et al*. Comparison of community‐wide, integrated mass drug administration strategies for schistosomiasis and soil‐transmitted helminthiasis: a cost‐effectiveness modelling study. Lancet Glob Health 2015; 3: e629–e638. 26385302 10.1016/S2214-109X(15)00047-9

[29] Doyen V , Corazza F , Nhu Thi H *et al*. Hookworm treatment induces a decrease of suppressive regulatory T cell associated with a Th2 inflammatory response. PLoS One 2021; 16: e0252921. 34111180 10.1371/journal.pone.0252921 PMC8191899

[30] Schmiedel Y , Mombo‐Ngoma G , Labuda LA *et al*. CD4+CD25hiFOXP3+ regulatory T cells and cytokine responses in human schistosomiasis before and after treatment with Praziquantel. PLoS Negl Trop Dis 2015; 9: e0003995. 26291831 10.1371/journal.pntd.0003995 PMC4546370

[31] Wilson S , Jones FM , Kenty LC *et al*. Posttreatment changes in cytokines induced by *Schistosoma mansoni* egg and worm antigens: dissociation of immunity‐ and morbidity‐associated type 2 responses. J Infect Dis 2014; 209: 1792–1800. 24357629 10.1093/infdis/jit826 PMC4017363

[32] Rajamanickam A , Munisankar S , Bhootra Y *et al*. Altered levels of memory T cell subsets and common γc cytokines in *Strongyloides stercoralis* infection and partial reversal following anthelmintic treatment. PLoS Negl Trop Dis 2018; 12: e0006481. 29795573 10.1371/journal.pntd.0006481 PMC5991401

[33] International Agency for Research on Cancer (IARC) . IARC monographs on the identification of carcinogenic hazards to humans: list of classifications. Lyon, France: World Health Organization; 1994. Available from: https://monographs.iarc.who.int/list-of-classifications. Accessed November 22, 2025

[34] Sabry Ael H , El‐Aal AA , Mahmoud NS , Nabil Y , Aziz IZ . An initial indication of predisposing risk of *Schistosoma mansoni* infection for hepatocellular carcinoma. J Egypt Soc Parasitol 2015; 45: 233–240. 26485842 10.12816/0017568

[35] Ishii A , Matsuoka H , Aji T *et al*. Parasite infection and cancer: with special emphasis on *Schistosoma japonicum* infections (Trematoda). A review. Mutat Res‐Fund Mol M. 1994; 305: 273–281. 10.1016/0027-5107(94)90247-x 7510038

[36] Ruiz‐Manzano RA , Palacios‐Arreola MI , Hernández‐Cervantes R *et al*. Potential novel risk factor for breast cancer: *Toxocara canis* infection increases tumor size due to modulation of the tumor immune microenvironment. Front Oncol 2020; 10: 736. 32547942 10.3389/fonc.2020.00736 PMC7272683

[37] Aragón‐Franco R , Ruiz‐Manzano RA , Nava‐Castro KE *et al*. Convergence between helminths and breast cancer: intratumoral injection of the excretory/secretory antigens of the human parasite *Toxocara canis* (EST) increase lung macro and micro metastasis. Front Immunol 2024; 15: 1332933. 38576624 10.3389/fimmu.2024.1332933 PMC10993691

[38] Hayes KS , Cliffe LJ , Bancroft AJ *et al*. Chronic *Trichuris muris* infection causes neoplastic change in the intestine and exacerbates tumour formation in APC min/+ mice. PLoS Negl Trop Dis 2017; 11: e0005708. 28650985 10.1371/journal.pntd.0005708 PMC5501682

[39] Maizels RM , McSorley HJ . Regulation of the host immune system by helminth parasites. J Allergy Clin Immunol 2016; 138: 666–675. 27476889 10.1016/j.jaci.2016.07.007 PMC5010150

[40] Hawerkamp HC , Fallon PG . Expelliarmus helminthus! Harry helminth and the goblet of Alarmins. Immunity 2022; 55: 575–577. 35417668 10.1016/j.immuni.2022.03.014

[41] Mariathasan S , Turley SJ , Nickles D *et al*. TGFβ attenuates tumour response to PD‐L1 blockade by contributing to exclusion of T cells. Nature 2018; 554: 544–548. 29443960 10.1038/nature25501 PMC6028240

[42] Machicado C , Marcos LA . Carcinogenesis associated with parasites other than *Schistosoma*, *Opisthorchis* and *Clonorchis*: a systematic review. Int J Cancer 2016; 138: 2915–2921. 26840624 10.1002/ijc.30028

[43] Salazar AM , Mendlovic F , Cruz‐Rivera M *et al*. Genotoxicity induced by *Taenia solium* and its reduction by immunization with calreticulin in a hamster model of taeniosis. Environ Mol Mutagen 2013; 54: 347–353. 23704053 10.1002/em.21782

[44] Herrera LA , Benita‐Bordes A , Sotelo J *et al*. Possible relationship between neurocysticercosis and hematological malignancies. Arch Med Res 1999; 30: 154–158. 10372452 10.1016/s0188-0128(98)00027-x

[45] Kazemi F , Moradi‐Sardareh H , Arjmand R , Tavalla M , Amari A , Cheraghian B . *Toxocara canis* increases the potential of breast cancer by reducing the expression of the P53 protein. Curr Mol Med 2024; 24: 335–343. 36959144 10.2174/1566524023666230320103506

[46] Szuba M , Stachera W , Piwko A *et al*. Geohelminths: use in the treatment of selected human diseases. Pathogens 2024; 13: 13. 10.3390/pathogens13080703 PMC11356798 39204303

[47] Ayelign B , Akalu Y , Teferi B , Molla MD , Shibabaw T . Helminth induced immunoregulation and novel therapeutic avenue of allergy. J Asthma Allergy 2020; 13: 439–451. 33116652 10.2147/JAA.S273556 PMC7548329

[48] Summers RW , Elliott DE , Urban JF Jr , Thompson R , Weinstock JV . Trichuris suis therapy in Crohn's disease. Gut 2005; 54: 87–90. 15591509 10.1136/gut.2004.041749 PMC1774382

[49] Croese J , O'Neil J , Masson J *et al*. A proof of concept study establishing *Necator americanus* in Crohn's patients and reservoir donors. Gut 2006; 55: 136–137. 16344586 10.1136/gut.2005.079129 PMC1856386

[50] Hunter MM , Wang A , Hirota CL , McKay DM . Neutralizing anti‐IL‐10 antibody blocks the protective effect of tapeworm infection in a murine model of chemically induced colitis. J Immunol 2005; 174: 7368–7375. 15905584 10.4049/jimmunol.174.11.7368

[51] Liu Q , Sundar K , Mishra PK *et al*. Helminth infection can reduce insulitis and type 1 diabetes through CD25‐ and IL‐10‐independent mechanisms. Infect Immun 2009; 77: 5347–5358. 19752032 10.1128/IAI.01170-08 PMC2786463

[52] Ryan SM , Ruscher R , Johnston WA *et al*. Novel antiinflammatory biologics shaped by parasite–host coevolution. Proc Natl Acad Sci USA 2022; 119: e2202795119. 36037362 10.1073/pnas.2202795119 PMC9457177

[53] Bhengu KN , Naidoo P , Singh R *et al*. Immunological interactions between intestinal helminth infections and tuberculosis. Diagnostics (Basel) 2022; 12: 12. 10.3390/diagnostics12112676 PMC9689268 36359526

[54] Akanksha K , Kumari A , Dutta O , Prasanth A , Deeba F , Salam N . Prevalence of soil‐transmitted helminth infections in HIV patients: a systematic review and meta‐analysis. Sci Rep 2023; 13: 11055. 37422549 10.1038/s41598-023-38030-y PMC10329649

[55] Salgame P , Yap GS , Gause WC . Effect of helminth‐induced immunity on infections with microbial pathogens. Nat Immunol 2013; 14: 1118–1126. 24145791 10.1038/ni.2736 PMC4955540

[56] de Grossi Oliveira AL , de Parreiras Jesus AC , Brito RMM *et al*. Serological evidence of soil‐transmitted helminth infections as a potential risk for severity in leprosy patients. Trop Med Int Health 2025; 30: 1115–1123. 40817887 10.1111/tmi.70020 PMC12501561

[57] Borkow G , Bentwich Z . HIV and helminth co‐infection: is deworming necessary? Parasite Immunol 2006; 28: 605–612. 17042932 10.1111/j.1365-3024.2006.00918.x

[58] Walson JL , Herrin BR , John‐Stewart GC . Deworming helminth co‐infected individuals for delaying HIV disease progression. Cochrane Database Syst Rev 2009; 3: CD006419. 10.1002/14651858.CD006419.pub3 PMC2871762 19588389

[59] Elias D , Mengistu G , Akuffo H , Britton S . Are intestinal helminths risk factors for developing active tuberculosis? Trop Med Int Health 2006; 11: 551–558. 16553939 10.1111/j.1365-3156.2006.01578.x

[60] Abate E , Belayneh M , Idh J *et al*. Asymptomatic helminth infection in active tuberculosis is associated with increased regulatory and Th‐2 responses and a lower sputum smear positivity. PLoS Negl Trop Dis 2015; 9: e0003994. 26248316 10.1371/journal.pntd.0003994 PMC4527760

[61] Elias D , Akuffo H , Pawlowski A , Haile M , Schön T , Britton S . *Schistosoma mansoni* infection reduces the protective efficacy of BCG vaccination against virulent *Mycobacterium tuberculosis* . Vaccine 2005; 23: 1326–1334. 15661380 10.1016/j.vaccine.2004.09.038

[62] Afolabi MO , Ale BM , Dabira ED *et al*. Malaria and helminth co‐infections in children living in endemic countries: a systematic review with meta‐analysis. PLoS Negl Trop Dis 2021; 15: e0009138. 33600494 10.1371/journal.pntd.0009138 PMC7924789

[63] Vieira‐Santos F , Leal‐Silva T , de Lima Silva Padrão L *et al*. Concomitant experimental coinfection by *Plasmodium berghei* NK65‐NY and *Ascaris suum* downregulates the Ascaris‐specific immune response and potentiates Ascaris‐associated lung pathology. Malar J 2021; 20: 296. 34210332 10.1186/s12936-021-03824-w PMC8248286

[64] Craig JM , Scott AL . Antecedent *Nippostrongylus* infection alters the lung immune response to *Plasmodium berghei* . Parasite Immunol 2017; 39: e12441. 10.1111/pim.12441 PMC5505811 28475238

[65] Helmby H . Gastrointestinal nematode infection exacerbates malaria‐induced liver pathology. J Immunol 2009; 182: 5663–5671. 19380813 10.4049/jimmunol.0803790 PMC2796717

[66] Natukunda A , Zirimenya L , Nassuuna J *et al*. The effect of helminth infection on vaccine responses in humans and animal models: a systematic review and meta‐analysis. Parasite Immunol 2022; 44: e12939. 35712983 10.1111/pim.12939 PMC9542036

[67] Apiwattanakul N , Thomas PG , Iverson AR , McCullers JA . Chronic helminth infections impair pneumococcal vaccine responses. Vaccine 2014; 32: 5405–5410. 25131738 10.1016/j.vaccine.2014.07.107

[68] Chen L , Liu W‐q , Lei J‐h *et al*. Chronic *Schistosoma japonicum* infection reduces immune response to vaccine against hepatitis B in mice. PLoS One 2012; 7: e51512. 23272112 10.1371/journal.pone.0051512 PMC3522692

[69] Gibbs LC , Oviedo JM , Ondigo BN , Fairfax KC . Maternal helminth infection causes dysfunctional B cell development in male offspring. J Immunol 2024; 213: 1157–1169. 39185897 10.4049/jimmunol.2400158 PMC11537230

[70] Ondigo BN , Muok EMO , Oguso JK *et al*. Impact of mothers' schistosomiasis status during gestation on children's IgG antibody responses to routine vaccines 2 years later and anti‐schistosome and anti‐malarial responses by neonates in Western Kenya. Front Immunol 2018; 9: 1402. 29967622 10.3389/fimmu.2018.01402 PMC6015899

[71] Zhu F , Liu W , Liu T *et al*. A new role for old friends: effects of helminth infections on vaccine efficacy. Pathogens 2022; 11: 1163. 36297220 10.3390/pathogens11101163 PMC9608950

[72] World Health Organization . World Health Organization: ending the neglect to attain the sustainable development goals: a road map for neglected tropical diseases 2021–2030. 2021.

[73] Muehlenbachs A , Bhatnagar J , Agudelo CA *et al*. Malignant transformation of *Hymenolepis nana* in a human host. N Engl J Med 2015; 373: 1845–1852. 26535513 10.1056/NEJMoa1505892

[74] Durrani MI , Basit H , Blazar E . Diphyllobothriasis (fish tapeworm infection). In: StatPearls. Treasure Island: StatPearls Publishing; 2025. 31082015

[75] Steinmann P , Keiser J , Bos R , Tanner M , Utzinger J . Schistosomiasis and water resources development: systematic review, meta‐analysis, and estimates of people at risk. Lancet Infect Dis 2006; 6: 411–425. 16790382 10.1016/S1473-3099(06)70521-7

[76] World Health Organization Global Health Observatory . World health organization global health observatory: schistosomiasis. 2023.

[77] CDC . About soil‐transmitted helminths. Available from: https://www.cdc.gov/sth/about/index.html.

[78] World Health Organization . World health organization: strongyloidiasis. Control of Neglected Tropical Diseases.

[79] Lashaki EK , Mizani A , Hosseini SA *et al*. Global prevalence of enterobiasis in young children over the past 20 years: a systematic review and meta‐analysis. Osong Public Health Res Perspect 2023; 14: 441–450. 38204424 10.24171/j.phrp.2023.0204 PMC10788413

[80] Ramharter M , Butler J , Mombo‐Ngoma G , Nordmann T , Davi SD , Zoleko Manego R . The African eye worm: current understanding of the epidemiology, clinical disease, and treatment of loiasis. Lancet Infect Dis 2024; 24: e165–e178. 37858326 10.1016/S1473-3099(23)00438-3

[81] Turhan N , Esendagli G , Ozkayar O , Tunali G , Sokmensuer C , Abbasoglu O . Co‐existence of *Echinococcus granulosus* infection and cancer metastasis in the liver correlates with reduced Th1 immune responses. Parasite Immunol 2015; 37: 16–22. 25319434 10.1111/pim.12152

[82] El‐Tonsy MM , Hussein HM , Helal TE‐S , Tawfik RA , Koriem KM , Hussein HM . *Schistosoma mansoni* infection: is it a risk factor for development of hepatocellular carcinoma? Acta Trop 2013; 128: 542–547. 23932944 10.1016/j.actatropica.2013.07.024

[83] Hamid HKS . *Schistosoma japonicum*–associated colorectal cancer: a review. Am J Trop Med Hyg 2019; 100: 501–505. 30560774 10.4269/ajtmh.18-0807 PMC6402928

[84] Pastille E , Frede A , McSorley HJ *et al*. Intestinal helminth infection drives carcinogenesis in colitis‐associated colon cancer. PLoS Pathog 2017; 13: e1006649. 28938014 10.1371/journal.ppat.1006649 PMC5627963

